# Ten-years trend of dengue research in Indonesia and South-east Asian countries: a bibliometric analysis

**DOI:** 10.1080/16549716.2018.1504398

**Published:** 2018-08-09

**Authors:** Ahmad Watsiq Maula, Anis Fuad, Adi Utarini

**Affiliations:** a Department of Biostatistics, Epidemiology and Population Health, Faculty of Medicine, Public Health and Nursing, Universitas Gadjah Mada, Yogyakarta, Indonesia; b Department of Health Policy and Management, Faculty of Medicine, Public Health and Nursing, Universitas Gadjah Mada, Yogyakarta, Indonesia

**Keywords:** Dengue, bibliometrics, PubMed, research topics analysis, network analysis

## Abstract

**Background**: Dengue fever is a mosquito-borne viral disease with high incidence in over 128 countries. WHO estimates 500,000 people with severe dengue are hospitalized annually and 2.5% of those affected die. Indonesia is a hyperendemic country for dengue with an increasing number of cases in the last decade. Unfortunately, the trends of Indonesian dengue research are relatively unknown.

**Objective**: This research aimed to depict bibliographic trends and knowledge structure of dengue publications in Indonesia relative to that of South-east Asia (SEA) from 2007 to 2016.

**Methods**: Bibliographic data were collected from PubMed filtered by Indonesia country affiliation. The annual growth rate of publication was measured and compared with neighborhood countries in the SEA region. Network analysis was used to visualize emerging research issues.

**Results**: About 1,625 dengue-related documents originated from SEA region, of which Indonesia contributed 5.90%. The publication growth rate in Indonesia, however, is the highest in ASEAN region (28.87%). Total citations for documents published from Indonesia was 980, with an average of 14 citations per publication and *h-index* of 16. Within the first five years, the main research topics were related to insect vector and diagnostic method. While insect vector remained dominant in the last five years, other topics such as disease outbreak, dengue virus, and dengue vaccine started emerging.

**Conclusion**: In the last 10 years, dengue publications’ growth from Indonesia in international journals improved significantly, despite less number of publications compared to other SEA countries. Efforts should be made to improve the quantity and quality of publications from Indonesia. The research topics related to dengue in Indonesia are in line with studies in SEA. Stakeholders and policy makers are encouraged to develop a roadmap for dengue research in the future.

## Background

As one of the neglected tropical diseases, dengue infection has been re-emerging. The number of dengue infection cases was estimated to be 390 million spread over 128 countries[1]. Although globally, only 3.2 million cases were reported to WHO in 2015 [2].

**Table 1.** Top 10 most published journal in Indonesia and ASEAN.

Indonesia					ASEAN				
		Publications					Publications		
No	Journal name	N	%	IF	No	Journal name	N	%	IF
1.	PLoS neglected tropical diseases	9	9.3	3.9	1.	PLoS neglected tropical diseases	141	8.7	3.9
2.	Acta medica Indonesiana	8	8.2	0.9	2.	PloS one	79	4.9	4.4
3.	PloS One	7	7.2	4.4	3.	The Southeast Asian journal of tropical medicine and public health	69	4.2	2.6
4.	Japanese journal of infectious diseases	5	5.1	1.4	4.	The American journal of tropical medicine and hygiene	68	4.2	2.7
5.	The Southeast Asian journal of tropical medicine and public health	5	5.1	2.6	5.	Antiviral research	41	2.5	4.9
6.	BMC infectious diseases	5	5.1	0.7	6.	Journal of virology	35	2.1	4.6
7.	The American journal of tropical medicine and hygiene	4	4.1	2.7	7.	BMC infectious diseases	32	1.9	0.7
8.	Infection, genetics and evolution	3	3.1	2.6	8.	Tropical biomedicine	29	1.8	0.7
9.	Mathematical biosciences	3	3.1	1.3	9.	The Journal of infectious diseases	24	1.5	6.3
10.	Bioinformation	3	3.1	0.8	10.	Vaccine	24	1.5	3.6
Total		52	51.5%			Total	542	33.3%	
Impact Factor Average		2.1				Impact Factor Average	3.4		

Asia-Pacific region has the highest burden among other regions in the world. The infection pattern has been increasing in semi-urban areas. Vector disease control problems such as unplanned urban development, poor water storage practices, and unsatisfactory sanitary conditions were the main triggering factors [2]. The South-East Asia (SEA) region is one of dengue endemic areas. In 2015, about 451,422 dengue cases of the total number of cases globally (14.11%), were from this region. Estimated 1.8 billion people in South-East Asia are at risk of dengue infection [2].

Dengue cases in Indonesia were first reported in 1968, since then the country was considered as hyperendemic area. In 2004–2010, Indonesia became the second country with the highest dengue cases after Brazil [3]. In 2015, the number of dengue cases in Indonesia hit 129,650 in 2015 [4]. Indonesia suffered the highest economic burden of dengue in SEA region in terms of aggregate cost at 323 million USD or about 34 per cent of the total regional economic burden of dengue, followed by Thailand (31%) and Malaysia (13%). However, Singapore spent the highest burden per capita (12.65 USD), followed by Malaysia (4.38 USD) and Thailand (4.34 USD) [5,6].

The current situation of the high burden of dengue cases in Indonesia and the SEA region coupled with the absence of effective treatment, and lack of comprehensive vector control and successful public health interventions remain a formidable challenge. Information related to dengue research and publications are available in the bibliographic database. Bibliometric studies, – research that was based on bibliographic data – have been conducted elsewhere to provide a mapping of the research and development activities on dengue [7]. This study aimed to depict bibliographic trends and knowledge structure of dengue publications in Indonesia relative to that of SEA from 2007 to 2016.

## Methods

### Data source

Bibliographical data related to dengue publications was retrieved in May 2017 from the PubMed database, developed by the National Library of Medicine (NLM) the USA. PubMed was selected since it is the main and specific database for biomedical literature consisting of 27 million publications. Compared to other bibliographic databases, such as Web of Science, Google Scholar, and Scopus, PubMed has the advantage of using standardized keywords for all documents stored in the database, ie the Medical Subject Heading (MeSH). Standard terms in MeSH was very useful for bibliometric analysis to depict the publication topics. In addition to PubMed, the Scopus database was also used to obtain information on related citations of documents.

### Study design

This study used a bibliometric analysis using methods proposed by previous studies [6–8]. A bibliometric analysis is a statistical method to analyze literature to depict the development trends of knowledge and other information (such as citation information, authorship, and keyword co-occurrence). It is also useful to provide progress or indicators of developmental trends in the science publications [9].

### Search strategy

The query used for the bibliometric analysis in PubMed database involved combining the keywords in the MeSH, Title, Abstract, Author Affiliation and the time period of 1 January 2007–31 December 2016.

A two-step query was conducted. At a global level, we used the keywords ‘dengue’[MeSH Terms] OR dengue[tiab] OR ‘dengue vaccines’[mesh] OR ‘dengue virus’[mesh] OR ‘severe dengue’[mesh]. The result of this first query was then filtered by SEA countries (Brunei Darussalam, Cambodia, Indonesia, Lao PDR, Malaysia, Myanmar, Philippines, Singapore, Thailand, and Viet Nam) based on information regarding author affiliation. We also considered country aliases name (such as Laos alias of Lao PDR and Burma alias of Myanmar) to filter the country affiliated publications.

**Figure 1.**
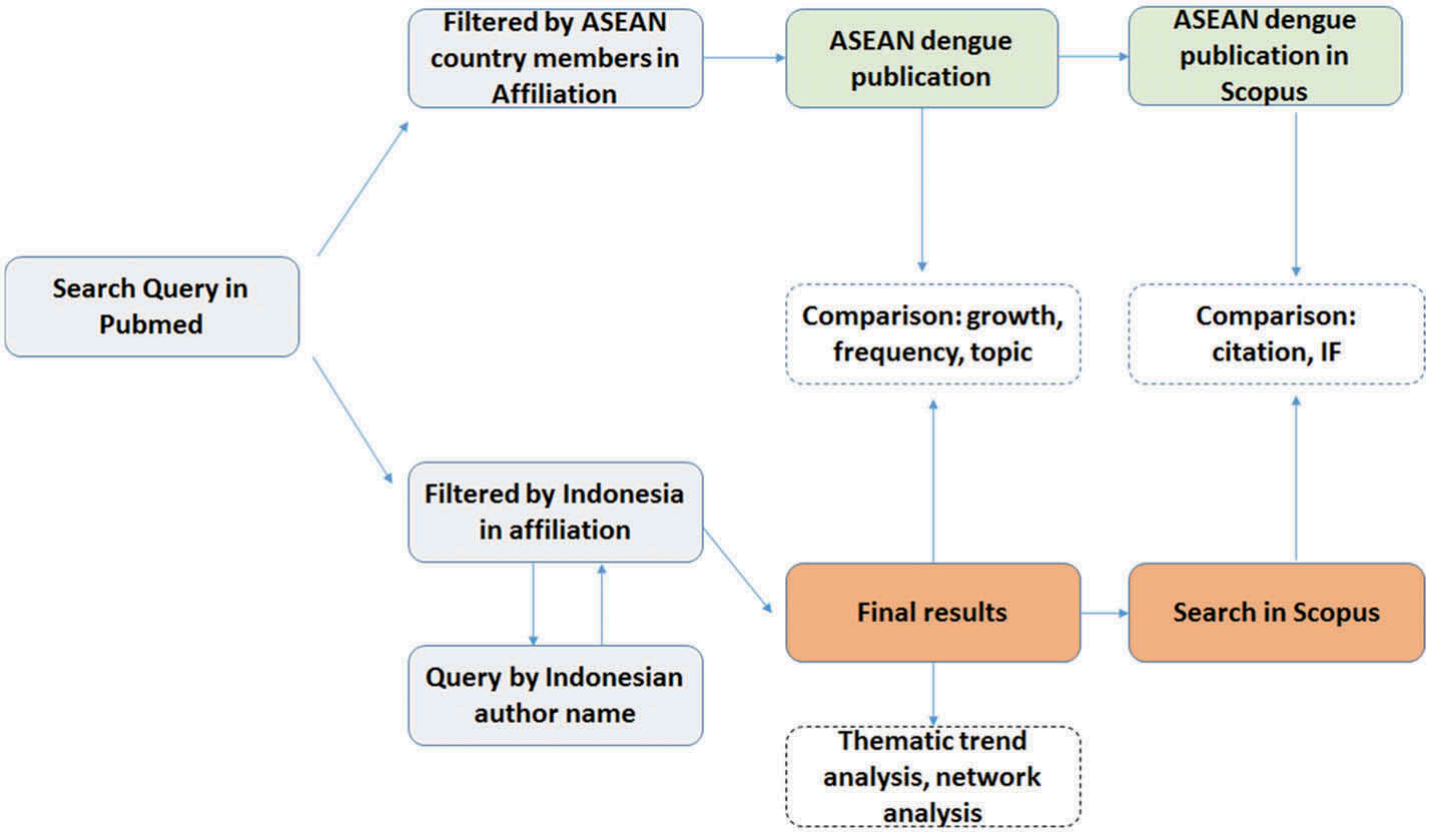
Bibliometric study strategy.

The variables included in the bibliometric analysis were: publication year, document type, the name of journals, impact factors (IF), author institutions, number of citations, *h-index*, and international collaboration. For further analysis, only dengue-related documents from Indonesia were extracted based on the author affiliation to Indonesia only. Medical Subject Heading (MeSH) in PubMed was used to get specific dengue related documents. MeSH is a controlled vocabulary created by the NLM to index bibliographical archives. Experienced indexers in NLM perform standard steps and procedures before assigning MeSH terms into any new article in Pubmed [10]. Journal impact factor for this study was retrieved from the *Journal Citation Reports (Thomson Reuters)* which is product from ISI Web of Knowledge. Thanks to the Pubmed ID (PMID), data linkage with Scopus database was performed to obtain citation number of each article (Figure 1).

### Data analysis

Data from PubMed were downloaded in XML format, then converted into Microsoft Excel™ using Pubmed2XL [11], to produce descriptive analysis on a number of publications per year, annual publication growth rate, list of most frequent journal used, and documents citation number.

To obtain a pattern of author collaborations and trends of evolving research during the period, we deployed a network analysis approach. A free bibliometric software, called VOSViewer version 1.6.6 [12] was utilized to visualize the evolving research trends and collaboration pattern. The bibliometric corpus was converted into a Social Network Analysis (SNA) graph using VOSViewer. SNA is an emerging method based on graph theory to map and measure the relationships and flows between entities (it could be people, groups, organizations, publication, terms, etc.) and other connected entities. An SNA graph will visualize the relationship pattern using nodes and edges. Node represents an entity such as country or a MeSH term. The bigger node indicates the higher occurrences of the term in the bibliometric data. Edge shows collaboration between countries and connections between topics (MeSH terms). The line will become thicker when co-occurrences of country and MeSH in document increase. A similar analysis was done for the pattern in co-authorship based on country affiliation data. Countries data were represented as node and collaboration of authors among the countries were shown as an edge (line). Normalization of network analysis was performed with LinLog/modularity to show co-authorship groups between countries.

Further analysis of topic trends was conducted based on the PubMed MeSH keywords to obtain the breadth of dengue research topics and their trends. Using VOSViewer, we performed a network analysis of co-occurrence of MeSH keywords in each document. MeSH was represented as a node, and the connection between MeSH in the same article was represented as an edge (line). The size of node became larger if MeSH keywords were frequently used. Grouping of research topics was visualized using normalization with LinLog/modularity analysis. We removed check tags MeSH, such as age groups, humans, animals, female/male that are not relevant to represent research topics [13].

Two measures were utilized to describe the quality of the publications, ie citation analysis and journal impact factor. Information about document citations was collected from the Scopus database. The citation information included article title, the name of the journal, author, and the number of citations for each article. The PubMed ID for all documents from Indonesia and ASEAN were used to perform searching in Scopus database. For the journal impact factor, the information was collected manually from the web pages of each related journal.

## Results

Globally, PubMed stores 10,908 dengue-related publications from 2007 to 2016. Within the last 10 years, the number of publication increased significantly (a three-fold increase). ASEAN country members contributed 1,625 (14.9%) published documents. Comparison among SEA countries revealed that Singapore was most productive (33.6%), followed by Thailand (32.2%), Malaysia (18.4%), Viet Nam (6.8%), and then Indonesia (5.6%). The *h-index* measures both productivity and citation impact of publications from each country. The five most productive SEA countries member had a *h-index* variance between 54 to 17, in which Singapore was the highest and Indonesia was the lowest among the top 5 SEA members.

**Figure 2.**
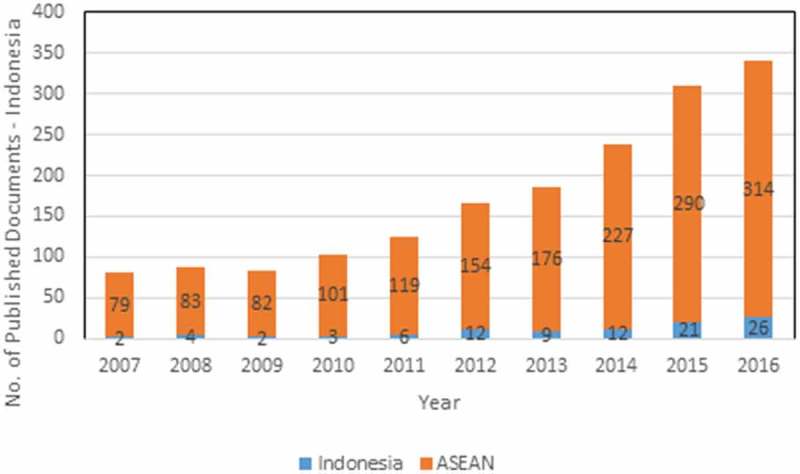
Annual number of dengue publications of Indonesia and ASEAN between 2007–2016.

Figure 2 shows that within the 10-year period, dengue publications from Indonesia increased 13 times (from only 2 publications in 2007 to 27 publications in 2016) compared with a five-fold increase for all countries in the ASEAN region. Although Indonesia ranked fifth in terms of total number of publications among the SEA countries, its publication growth rate (28.9%) was the highest in the region.

Journal Impact Factor (JIF) entails a highly controversial issue although widely used [14]. It is generally admitted as an imperfect parameter to measure the quality of the articles. Unfortunately, no better solution is available to measure scientific performance[15]. In Table 1, we compared JIF of the top 10 journals publishing dengue research from Indonesia and SEA countries. JIF of the top 10 journals publishing Indonesian dengue articles are ranging from 0.7 to 4.4. These 10 journals published 52 articles or 51.5% of the total Indonesian dengue publications. Impact Factor of the top 10 journals publishing dengue research from SEA region are not so different. They are ranging from 0.7 to 4.9 and published 542 articles or 33.3% from the total. However, average IF of journals publishing Indonesian dengue articles are lower (JIF = 2.1) than those from SEA region (JIF = 3.4)

The most cited publications appeared in journals with high IF of above 10. Two articles published by *The Lancet* journal (IF = 44.002) and *New England Journal of Medicine* (IF = 72.406) were cited by 402. Journals with lower IF were less cited, with a similar total number of citations (about 200 citations) among journals with IF below 2, between 2–4 and above 4. In addition, articles with the highest number of citations were multi-authored articles by authors from several countries.

**Figure 3.**
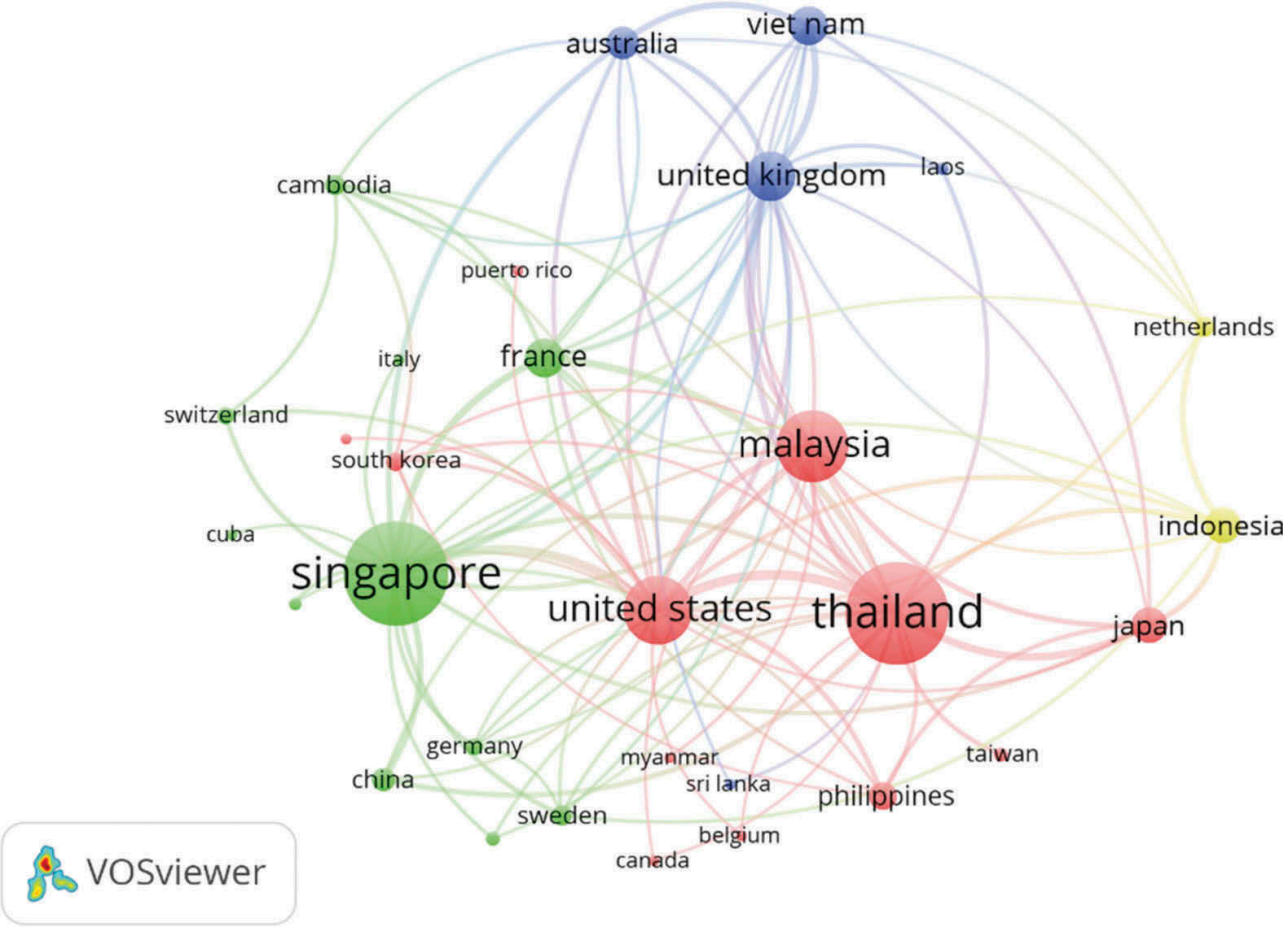
Southeast Asia countries co-authorship network related to dengue publication.

Social network analysis in Figure 3 displayed co-authorship pattern of SEA researchers on dengue. Four clusters of co-authorship collaboration were identified. The biggest cluster (in red) is led by Thailand, followed by the USA, Malaysia and the remaining countries. The second prominent cluster (green) is led by Singapore and is followed by other collaborating countries. Indonesia was belonging to the smallest cluster (yellow) showing that Indonesian authors tended to collaborate more with the Netherlands, Japan and USA.

The closer distance between nodes and or thicker edge connecting nodes indicate higher intensity of collaboration between authors from the respected countries. In each cluster SEA country seems to have convenient partner for research collaboration from the developed countries out of the SEA region. The graph showed that co-authorship collaboration among the SEA countries was limited.

The three most productive research institutions in Indonesia were University of Indonesia (Jakarta), Airlangga University (Surabaya) and Padjadjaran University (Bandung), while foreign institution partners that frequently were collaborating with Indonesia was Radboud University (Netherlands), followed by Mahidol University (Thailand) and Osaka University (Japan).

During the latest five-year period (2012–2016), we found that the major topics of dengue research in Indonesia were similar to that in the SEA countries based on 20 MeSH keywords most frequently used. About half of them were similar in Indonesia and SEA countries, such as dengue virus, and the insect vector (*Aedes*).

**Figure 4.**
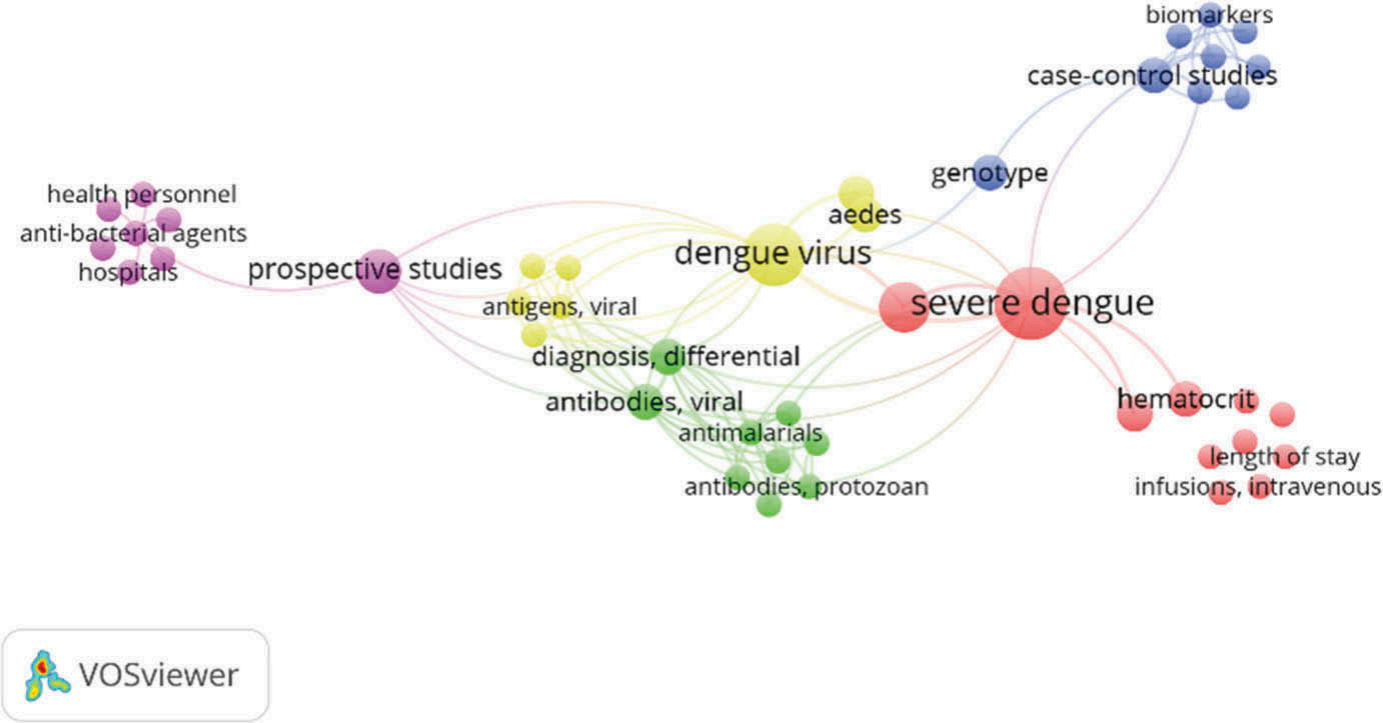
Social network analysis of dengue research topics from Indonesia (2012–2016).

**Figure 5.**
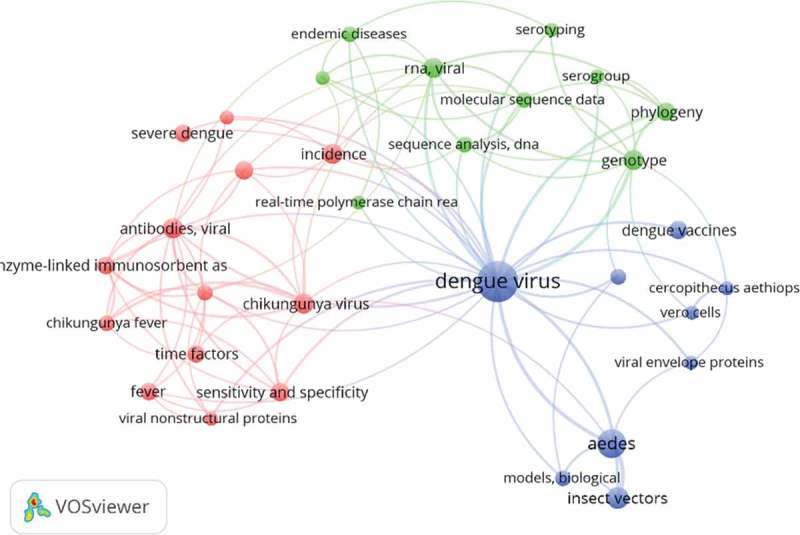
Social network analysis of dengue research topics from Indonesia (2012–2016).

**Figure 6.**
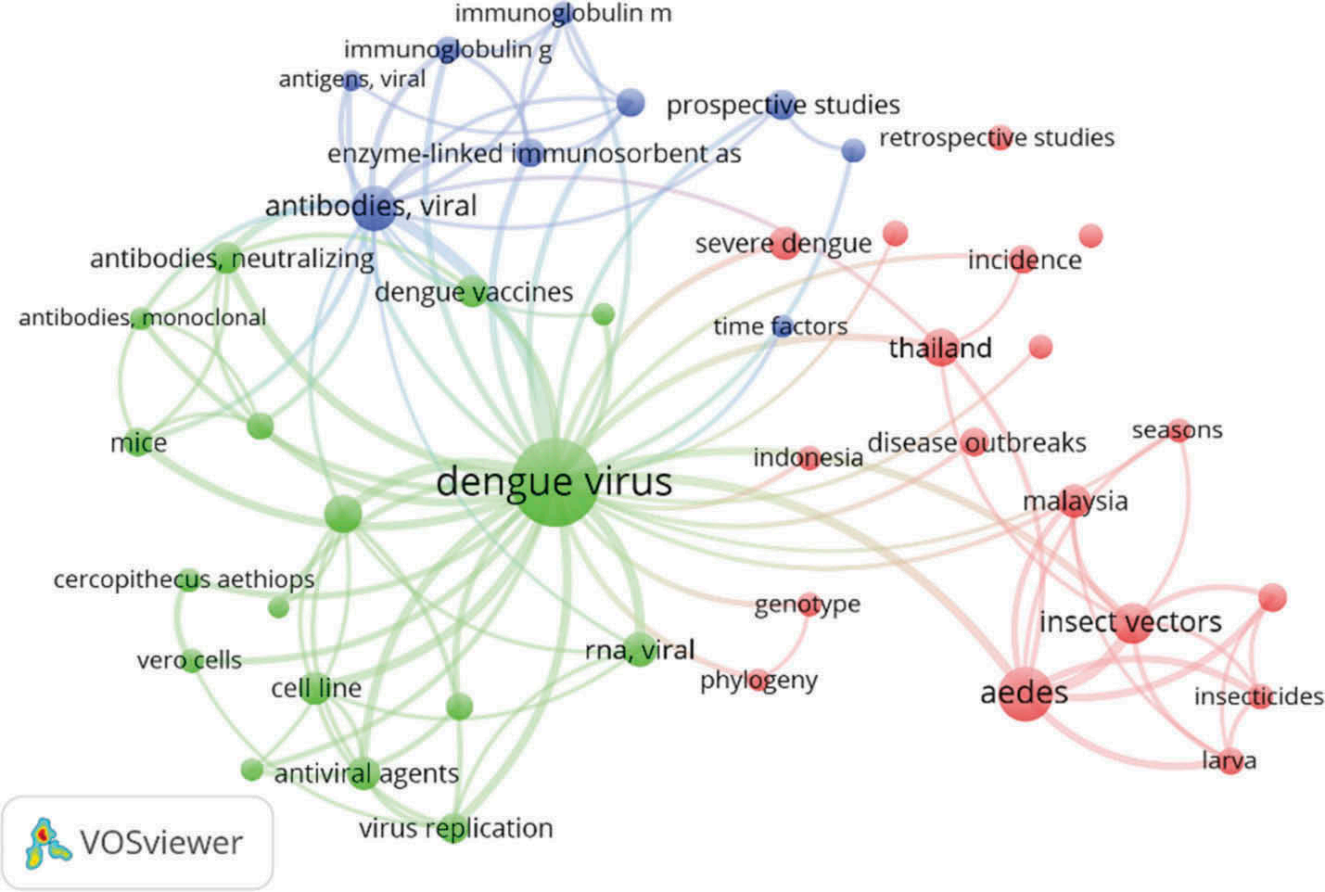
Social network analysis ASEAN research topics.

However, trends of the dengue-related publication topics changed over the first five-year period. In 2007–2011, the most frequent research topics were intravenous therapy, *Aedes* mosquito vectors, and diagnostic methods, especially the development of biomarkers. Then in the last period (2012–2016), new topics such as analysis of dengue virus structure, epidemiological studies related to dengue and dengue vaccines started emerging. Analysis of the structure of dengue virus revealed a close relationship with the development of dengue vaccines (Figures 4, 5). Further analysis using the social network analysis at the SEA region showed that there were some topics that were not frequently used in Indonesia (such as antiviral agents and epidemiological studies) (Figure 6).

Figures 4–6 explained the co-occurrence of keywords in a journal article. Figures 4, 5 visualized Indonesian co-occurrence of keywords (topics) in the first and second five-year periods. Comparison of Figures 4 and 5 revealed a shift of trends of dengue-related publication topics. For illustration, in 2007–2011, the most frequent research topics were intravenous therapy, Aedes mosquito vectors and diagnostic methods, especially the development of biomarkers. Then in the latest period (2012–2016), new topics such as analysis of dengue virus structure, epidemiological studies related to dengue and dengue vaccines started emerging. Severe dengue that was very prominent in the first period (Figure 4) became less evident in the next five-year period (Figure 5). Interestingly, the pattern in Figure 5 was qualitatively similar to Figure 6, indicating that the trend of research topics in Indonesia in the latest five-year period (2012–2016) was following a similar pattern in the SEA countries based on 20 MeSH keywords most frequently used. About half of them were similar in Indonesia and SEA countries, such as dengue virus, and the insect vector (*Aedes*). Some topics were not frequently used in Indonesia, such as antiviral agents and epidemiological studies (Figure 6).

## Discussion

This study shows significant growth of dengue publications by authors from Indonesia and the SEA region. Globally, dengue cases continuously increase due to various factors such as viral evolution, climate change, human mobility, and settlements [16,17]. In the last decade, research funding on dengue arisen [18].

From 2007 to 2011, dengue research and development funding increased by 4.7% [19]. In the early part of this period, dengue research was mostly funded by the governments, but then gradually, the private sector began to take over research funding for dengue. Since 2012, the pharmaceutical industry has spent more funds, especially for the development of dengue therapy and vaccine [20]. This trend creates markets for the private sector in developing countries, especially the pharmaceutical industry. Indonesia as one of the dengue endemic country started to publish more articles over the 10 years period because of this funding improvement. Changes in research funding also affect the development of research topics, ie a shift from dengue diagnosis and epidemiology to therapy and vaccines, which is a new trend emerging since 2012.

International co-authorship collaboration between ASEAN member countries occurred less than collaboration with countries outside the SEA region. This trend was probably due to the knowledge hubs related to prior education (ie authors from SEA or Indonesia received education in the developed country), and the need for more advanced facilities or research support, where the facilities are mostly owned by institutions in the developed countries. Similar findings occurred for publications on infectious disease such as tuberculosis and malaria [21].

Despite increasing number of publications applying bibliometrics analysis, the method has weaknesses related to the indicators used for assessing publication quality. Impact factor is suitable to depict quality of the journal, but it is less reliable to indicate quality of articles published. Journal IF particularly has advantage as easily available to access and widely used. However, it does not reflect the quality of each article in the journal. On the other side, number of citation of each article was sometime does not reflects the quality of the article [22].

Use of citation analysis to obtain article quality is sometimes unreliable because recently published articles have lower probabilities of being cited. Author’s behaviors to cite other article were motivated by on various reasons, for example, correct methodology and useful results of the study. However, authors sometime even cite other paper due to the opposite reasons, for example flawed method and results. Therefore, it could not be generated as an indicator of good article. It can only measure whether the paper was useful to other authors for writing their own papers [23].

Since bibliometric analysis is frequently use articles that only indexed in PubMed, Scopus, and Web of Science, articles from Indonesia or other countries local journal could not be included in this study. There are some problems to perform bibliometric study using local journal. First, in searching for specific articles, local language gives some obstacle to find article for analysis. Second, in Indonesia some local journal is not indexed in one portal, it will be time wasting activity to find the journal one by one. Indonesia started to index all journals in one portal called SINTA, it is good step to facilitating journal and author to improve publications in Indonesia. Other developing country could follow this method to make author easier to submit journal but recognized locally by the country.

## Conclusions

The growth of dengue publications from Indonesia in international journals improved significantly, despite less number of publications compared to other SEA countries. Both the quantity and quality of publications should be improved through multiple strategies. These vary from structured training interventions, provision of learning resources, availability of publication writing coach for authors, supported by academic atmosphere and regulations that promote journal publications. The research topics related to dengue in Indonesia are in line with studies in SEA, however, some specific topics on potential therapy, cost-benefit analysis in health facility level and prevention and control need to be further encouraged. Likewise, epidemiological studies using various study design. Stakeholders and policy makers are encouraged to develop a roadmap for dengue research in the future.

## Paper context

Dengue publication and research situation study is very important, especially in endemic areas. This study depicts the dengue research and publication situation in SEA region and Indonesia over the past 10 years. The journals’ selection for publication becomes very important to improve the quality of dengue publications in Indonesia specifically. In addition, the selection of research topics should also be considered in order to keep up with global level research topics.

## References

[1] Bhatt S, Gething PW, Brady OJ, et al The global distribution and burden of dengue. Nature. 2013;496:504–8. 23563266 10.1038/nature12060 PMC3651993

[2] WHO Integrating neglected tropical diseases in global health and development fourth WHO report on neglected tropical diseases. WHO [Internet]. 2017; Available from: http://www.who.int/neglected_diseases/resources/9789241565448/en/

[3] WHO Global strategy for dengue prevention and control 2012–2020. WHO Library Cataloguing-in-Publication Data, Switzerland. 2012 Sep 4. Available from: http://apps.who.int/iris/bitstream/10665/75303/1/9789241504034_eng.pdf

[4] Kementerian Kesehatan Profil kesehatan Indonesia 2015 [Internet]. Kementeri. Kesehat. Republik Indones. 2015 Available from: http://www.depkes.go.id/resources/download/pusdatin/profil-kesehatan-indonesia/profil-kesehatan-indonesia-2014.pdf

[5] Caballero-Anthony M, Cook ADB, Amul GGH, et al Health governance and dengue in Southeast Asia. Available from https://www.rsis.edu.sg/wp-content/uploads/2015/06/NTS-Report-No-2-10June2015.pdf

[6] Stanaway JD, Shepard DS, Undurraga EA, et al The global burden of dengue: an analysis from the global burden of dengue study 2013. Lancet Infect Dis. 2016;16:712–723. 26874619 10.1016/S1473-3099(16)00026-8 PMC5012511

[7] Zyoud SH. Dengue research: A bibliometric analysis of worldwide and Arab publications during 1872–2015. Virol J. 2016;13:78. 27154247 10.1186/s12985-016-0534-2 PMC4859974

[8] Mota FB, Fonseca BDP, Galina AC, et al Mapping the dengue scientific landscape worldwide: A bibliometric and network analysis. Mem Inst Oswaldo Cruz. 2017;112:354–363. 28443981 10.1590/0074-02760160423 PMC5398162

[9] Thompson DF, Walker CK A descriptive and historical review of bibliometrics with applications to medical sciences Pharmacotherapy. 2015;35:551–559. 10.1002/phar.1586 25940769

[10] The medline indexing process: determining subject content. [cited 2018 7 13]. Available from: https://www.nlm.nih.gov/bsd/disted/meshtutorial/principlesofmedlinesubjectindexing/theindexingprocess/

[11] Isaak D PubMed2XL (version 2.01). J. Med Libr Assoc [Internet]. 2016;104:92–94. Available from: http://www.ncbi.nlm.nih.gov/pmc/articles/PMC4722658/

[12] Van Eck NJ, Waltman L Software survey: Vosviewer, a computer program for bibliometric mapping. Scientometrics. 2010;84:523–538. 20585380 10.1007/s11192-009-0146-3 PMC2883932

[13] NIH Check Tags [Internet]. cited 2017 12 1 Available from: https://www.nlm.nih.gov/bsd/indexing/training/CHK_010.html

[14] Garfield E The history and meaning of the journal impact factor. JAMA 2006;295(1):90–93. 10.1001/jama.295.1.90 16391221

[15] Hoeffel C Journal impact factors. Allergy. 1998;53:1225. 10.1111/j.1398-9995.1998.tb03848.x 9930604

[16] Murray NEA, Quam MB, Wilder-Smith A Epidemiology of dengue: past, present and future prospects. Clin Epidemiol. 2013;5:299–309. 23990732 10.2147/CLEP.S34440 PMC3753061

[17] Wilder-Smith A, Gubler DJ Geographic expansion of dengue: the impact of international travel. Med Cli North Am. 2008;92(6):1377–1390. 10.1016/j.mcna.2008.07.002 19061757

[18] Wilder-Smith A, Schwartz E Dengue in Travelers. N. Engl. J. Med. Internet. 2005;353:924–932. Available from http://www.nejm.org/doi/abs/10.1056/NEJMra041927 16135837 10.1056/NEJMra041927

[19] Horstick O, Tozan Y, Wilder-Smith A Reviewing dengue: still a neglected tropical disease? PLoS Negl Trop Dis. 2015;9(4):e0003632. 10.1371/journal.pntd.0003632 PMC4415787 25928673

[20] G-FINDER Policy cure research [Internet]. 2017 [cited 2007 7 20]. Available from: https://gfinder.policycures.org/PublicSearchTool/

[21] Malbas V Mapping the collaboration networks of biomedical research in Southeast Asia. PeerJ PrePrints 2015;3:e936v1.

[22] Durieux V, Gevenois PA Bibliometric indicators: quality measurements of scientific publication. Radiology. 2010;5;255:342–351. 20413749 10.1148/radiol.09090626

[23] Belter CW Bibliometric indicators: opportunities and limits. J Med Libr Assoc. 2015;10;103:219–221. 26512227 10.3163/1536-5050.103.4.014 PMC4613388

